# Thermal Conductivity Measurement of an Electron-Beam Physical-Vapor-Deposition Coating

**DOI:** 10.6028/jres.108.014

**Published:** 2003-04-01

**Authors:** A. J. Slifka, B. J. Filla

**Affiliations:** National Institute of Standards and Technology, 325 Broadway, Boulder, CO 80303

**Keywords:** EB-PVD, thermal barrier coating, thermal conductivity, yttria-stabilized zirconia

## Abstract

An industrial ceramic thermal-barrier coating designated PWA 266, processed by electron-beam physical-vapor deposition, was measured using a steady-state thermal conductivity technique. The thermal conductivity of the mass fraction 7 % yttria-stabilized zirconia coating was measured from 100 °C to 900 °C. Measurements on three thicknesses of coatings, 170 μm, 350 μm, and 510 μm resulted in thermal conductivity in the range from 1.5 W/(m·K) to 1.7 W/(m·K) with a combined relative standard uncertainty of 20 %. The thermal conductivity is not significantly dependent on temperature.

## 1. Introduction

Thermal barrier coatings are used in gas turbine engines on hot surfaces of structural components to extend the lifetimes of these components. Although the adhesion of these coatings to superalloy substrates is of primary importance, the thermal conductivity of the coatings must also be known for design purposes. In addition, coatings with stable microstructure and lower thermal conductivity would provide longer engine lifetimes. Values typically found in the literature for thermal conductivity of yttria-stabilized (mass fraction 7 % to 8 %) zirconia produced by electron-beam physical vapor deposition (EB-PVD) range from 1.4 W/(m·K) to 1.7 W/(m·K) [1, 2].

Six specimens of 7 % mass fraction yttria-stabilized zirconia coating produced by EB-PVD on nickel-based superalloy substrates have been measured for thermal conductivity. The coatings, designated PWA 266,^1^ measured here had three thicknesses. Two sets of specimens each with nominal coating thicknesses of 170 μm, 350 μm, and 510 μm on 3 mm substrates were measured. In addition, the superalloy substrate material, designated PWA 1484, was measured using three thicknesses of 2 mm, 3 mm, and 7 mm. All of the specimens were provided by a commercial manufacturer of gas turbine engines. Thermal conductivity measurements were done over a range of temperature from approximately 100 °C to 900 °C for the coating and from 100 °C to 800 °C for the superalloy.

## 2. Measurement and Data

**Fig. 1:**
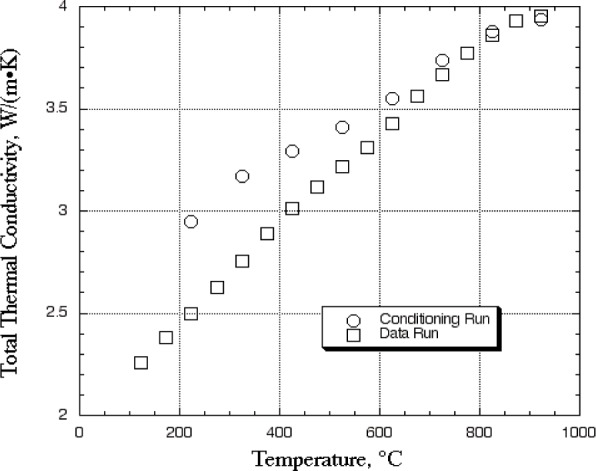
Raw thermal conductivity data for one of the 350 μm thick coated specimens after polishing to 0.7 μm average surface roughness.

A one-sided guarded-hot-plate apparatus was used for measurement of thermal conductivity. We had previously measured a plasma-sprayed coating using this method [3]. Details of this measurement system can be found in the literature [3, 4]. The raw data from the measurement includes the effect of the substrate material and the interfacial thermal resistances between the sensor plates and the specimen. Figure 1 shows a typical measurement, given as total thermal conductivity as a function of temperature. We measured three specimens in the as-received condition, but the inter-facial thermal resistance between the coating and sensor plate was too high for reliable thermal conductivity measurement due to the roughness of the coatings. We then polished all six specimens to nominally the same surface roughness (Ra), 0.7 μm. By using two specimens of different thickness, the thermal conductivity of the coating can be determined as long as the surface finishes of the two specimens are similar and the thicknesses of the substrates are within a few percent. Since all of the coated specimens are produced using the same process and process parameters, the interfacial thermal resistance between the coating and substrate should be consistent between specimens and its effect is essentially eliminated when using two or more specimens for this measurement technique. In order to reduce the uncertainty of the measurement, we have measured the thermal conductivity of the nickel-based superalloy substrate material, and we subtract the effect of this thermal resistance when determining the thermal conductivity of the coating.

**Fig. 2:**
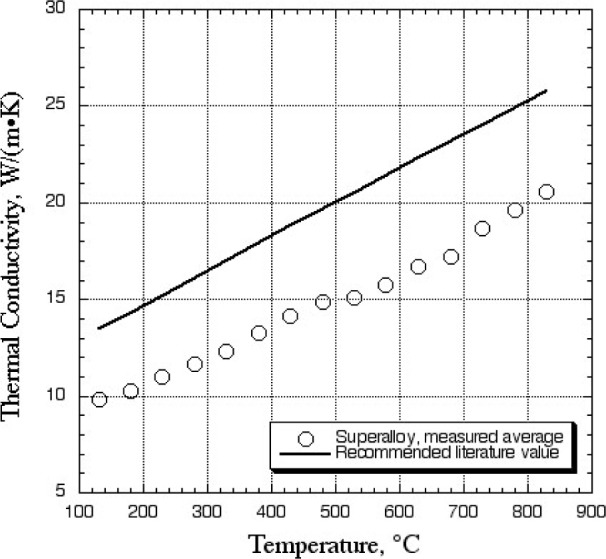
Measured thermal conductivity compared with recommended literature values for a similar class of alloy.

The superalloy material is a nickel-based alloy with a composition including nominally mass fractions 60 % Ni, 5 % Cr, 10 % Co, and a number of additional alloying elements. This superalloy material is in the form of a single crystal, and as such is used as turbine blade material in high-performance commercial gas-turbine engines. The superalloy came from the manufacturer with a proprietary surface oxidation to minimize changes to the surface and therefore the interfacial thermal contact with the sensor plates during measurement. The average surface finish of the material was 0.1 μm. Due to the highly alloyed nature of the material, the thermal conductivity is low when compared to other common metals. However, the trend of thermal conductivity, as shown in Fig. 2, is commensurate with that of other nickel + chromium + (other alloy elements) materials. Recommended literature values for this general classification of alloys is shown in the figure for comparison [5]. Like the coating measurements, three thicknesses were measured to determine both thermal conductivity of the material and inter-facial thermal resistance between the specimen and sensor plates. These two quantities require two measurements on specimens of different thickness. The third measurement is made in order to assure measurement reliability. The combined standard uncertainty of the apparatus under design conditions is 5 % [4]. Even a small amount of oxidation during measurement can cause additional uncertainty. The measurements of the three thicknesses, when combined as pairs for analysis, fall within 10 % of each other. Based on other measurements of oxidizing metals, a combined relative standard uncertainty for this measurement of 10 % is not unusual. The average result of these measurements is lower than the recommended literature values for nickel + chromium + (other alloy elements). However, the composition of PWA 1484 is lower in nickel and chromium than most alloys in this literature classification. Within the literature values, alloys with low nickel and chrome showed the same thermal conductivity as that of the superalloy measured here [5].

**Fig. 3:**
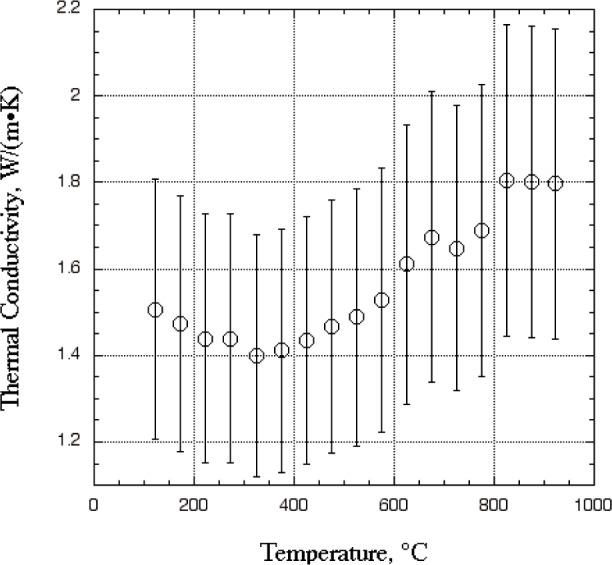
Thermal conductivity of the ceramic coating (average data) with 20 % relative standard uncertainty intervals.

Figure 3 shows data for the average thermal conductivity of all six coated specimens. The uncertainty bars shown are for a 20 % relative standard uncertainty. The combined standard uncertainty of the apparatus is 5 % [4]. However, the apparatus was designed for measuring monolithic ceramic materials of greater thickness. Due to the inherent interfacial thermal resistance between the sensor plates and the specimen, the reliability of the measurement is influenced by the ratio of the thermal resistance due to the specimen and the thermal resistance of the contact between the specimen and sensor plates. In general, the thermal resistance due to the specimen should be at least four times greater than that of the interfacial thermal resistance between the specimen and sensor plates. However, the small thicknesses of some of the specimens results in a low thermal resistance of the coatings relative to that of the specimen/sensor-plate interface. This factor significantly increases the uncertainty of the measurement of thermal conductivity of the coating vs temperature.

## 3. Discussion and Conclusions

**Fig. 4:**
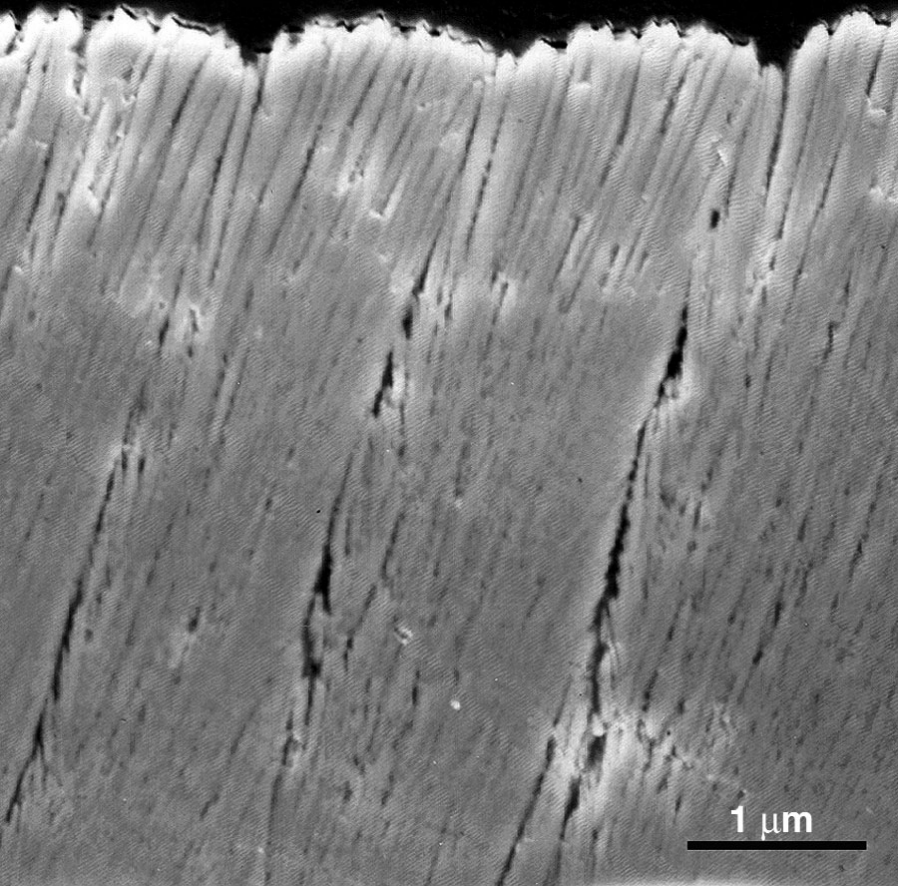
SEM image of an electron-beam directed-vapor-deposition coating showing micro-structure similar to the EB-PVD coating measured here. (Image courtesy of D. D. Hass)

The average thermal conductivity of PWA 266 was measured over temperatures ranging from approximately 100 °C to 900 °C. The measured results vary from 1.5 W·m^−1^·K^−1^ to 1.7 W·m^−1^·K^−1^ with a relative uncertainty range of 20 % at each temperature. The results are relatively independent of temperature. Due to the fine microstructure and pororosity of yttria-stabilized EB-PVD coatings, the thermal conductivity is expected to be independent of temperature. Even though EB-PVD coatings grow in a directional manner, resulting in a high degree of texture, they do not exhibit thermal behavior normally associated with texture. Figure 4 is an SEM image of an EB-PVD coating showing fine structure, which promotes geometric scattering of phonons. Measurements on similar coatings show a three-level structure of porosity that can result in significant reduction of thermal conductivity from that of sintered polycrystalline material [2]. The large amount of scattering due to the disorder of the stabilized zirconia lattice, combined with the geometric scattering due to a large population of other defects causes these coatings to have a lattice thermal conductivity that is not sensitive to temperature.

## Footnotes

1 Certain commercial equipment, instruments, or materials are identified in this paper to foster understanding. Such identification does not imply recommendation or endorsement by the National Institute of Standards and Technology, nor does it imply that the materials or equipment identified are necessarily the best available for the purpose.
